# Response Inhibition during Cue Reactivity in Problem Gamblers: An fMRI Study

**DOI:** 10.1371/journal.pone.0030909

**Published:** 2012-03-30

**Authors:** Ruth J. van Holst, Mieke van Holstein, Wim van den Brink, Dick J. Veltman, Anna E. Goudriaan

**Affiliations:** 1 Academic Medical Center, Department of Psychiatry, University of Amsterdam, Amsterdam, The Netherlands; 2 Amsterdam Institute for Addiction Research, Amsterdam, The Netherlands; 3 VU University Medical Center, Department of Psychiatry, Amsterdam, The Netherlands; 4 Donders Centre for Cognitive Neuroimaging, Radboud University, Nijmegen, The Netherlands; The University of Melbourne, Australia

## Abstract

Disinhibition over drug use, enhanced salience of drug use and decreased salience of natural reinforcers are thought to play an important role substance dependence. Whether this is also true for pathological gambling is unclear. To understand the effects of affective stimuli on response inhibition in problem gamblers (PRGs), we designed an affective Go/Nogo to examine the interaction between response inhibition and salience attribution in 16 PRGs and 15 healthy controls (HCs).

Four affective blocks were presented with Go trials containing neutral, gamble, positive or negative affective pictures. The No-Go trials in these blocks contained neutral pictures. Outcomes of interest included percentage of impulsive errors and mean reaction times in the different blocks. Brain activity related to No-Go trials was assessed to measure response inhibition in the various affective conditions and brain activity related to Go trials was assessed to measure salience attribution.

PRGs made fewer errors during gamble and positive trials than HCs, but were slower during all trials types. Compared to HCs, PRGs activated the dorsolateral prefrontal cortex, anterior cingulate and ventral striatum to a greater extent while viewing gamble pictures. The dorsal lateral and inferior frontal cortex were more activated in PRGs than in HCs while viewing positive and negative pictures. During neutral inhibition, PRGs were slower but similar in accuracy to HCs, and showed more dorsolateral prefrontal and anterior cingulate cortex activity. In contrast, during gamble and positive pictures PRGs performed better than HCs, and showed lower activation of the dorsolateral and anterior cingulate cortex.

This study shows that gambling-related stimuli are more salient for PRGs than for HCs. PRGs seem to rely on compensatory brain activity to achieve similar performance during neutral response inhibition. A gambling-related or positive context appears to facilitate response inhibition as indicated by lower brain activity and fewer behavioural errors in PRGs.

## Introduction

Pathological gambling is characterized by persistent and recurrent maladaptive gambling behaviour (American Psychiatric Association 2003). Up to 50% of problem gamblers report that direct presentation of gambling stimuli is a trigger to gamble (Grant and Kim 2001). However, the mechanisms underlying this recurrent maladaptive gambling behaviour are still unclear.

An influential and empirically grounded neurobiological model for substance dependence, the Impaired Response Inhibition and Salience Attribution (I-RISA) model, postulates that repeated drug use triggers a series of adaptations in neuronal circuits involved in memory, motivation, and cognitive control. If an individual has used drugs, memories of this event are stored as associations between the stimulus and the elicited positive (pleasant) or negative (aversive) experiences, facilitated by dopaminergic activation caused by the drug of abuse. This results in an enhanced (and long-lasting) salience for the drug and its associated cues at the expense of decreased salience for natural reinforcers [1]. In addition, the I-RISA model assumes loss of control (disinhibition) over drugs due to enhanced salience and pre-existing deficiencies, which renders individuals suffering from addictive disorders vulnerable to relapse into addictive behaviour. Although the I-RISA model is based on findings in substance dependent subjects, converging evidence suggests that this model could also explain the development and course of pathological gambling [2]–[4].

Enhanced salience attribution towards gambling cues has consistently been reported in problem gamblers. Functional magnetic resonance imaging (fMRI) studies in problem gamblers compared to controls investigating salience attribution (i.e. cue reactivity) towards gambling pictures have found enhanced Blood Oxygen Level-Dependent (BOLD) responses in the amygdala, cingulate cortex, dorsolateral prefrontal cortex (DLPFC) and ventrolateral prefrontal cortex (VLPFC) [5], [6], similar to the enhanced BOLD responses to drug-related pictures or movies in alcohol and drug dependent subjects [7]–[9]. Diminished sensitivity towards monetary wins and losses as observed in substance dependent disorders [7], [10], [11] has also been reported in problem gamblers. For example, in fMRI paradigms where participants experienced small monetary gains and losses, problem gamblers showed attenuated responses in the ventral striatum, and ventromedial and ventral lateral prefrontal cortex compared to controls [12]–[14]. However, the majority of problem gamblers are used to play with large amounts of money, which could also explain the attenuated response to winning or losing small amounts of money. Evidence of diminished sensitivity towards non-monetary cues in gamblers should therefore be tested, for example with positive or negative affective pictures which are also known to recruit salience/motivational circuitry, including amygdala, striatum, and orbitofrontal cortex [15].

Cognitive control and impulse regulation are critically dependent on intact prefrontal cortex functioning, in particular the inferior frontal cortex (IFC), anterior cingulate (ACC) and DLPFC [16]–[19]. Diminished IFC, ACC and DLPFC activity associated with impaired response inhibition has been reported in individuals with a substance use disorder [20]–[22]. In contrast, some other studies found similar response inhibition performance in substance dependent groups and healthy controls, together with increased activity in IFC, ACC and DLPFC in the substance dependent groups [23], [24]. These latter findings have been interpreted as indicative of a compensatory brain response in substance dependent individuals to achieve a similar level of performance as controls.

Impaired response inhibition has been reported in behavioural studies in problem gamblers, e.g., increased cognitive interference on the Stroop task, and diminished inhibition in stop-signal tasks [25], [26]. However, similar to the literature in substance use disorders, some studies failed to observe behavioural differences between problem gamblers and healthy controls [27]–[29]. The mixed results in studies on response inhibition in problem gamblers may be explained by the presence of comorbid conditions or differences in gambling problem severity in these studies [e.g., 30]. Alternatively, the distributed cortical and subcortical network supporting efficient response inhibition, such as the DLPFC, may be functionally intact, with impaired error processing being responsible for diminished response inhibition [31]. The two neuroimaging studies on this topic to date, indicate diminished ventral lateral prefrontal cortex activity in PRGs compared to controls during response inhibition on a Stroop task between problem gamblers and controls [32] and diminished responsiveness of the dorsomedial prefrontal cortex during a stop-signal response inhibition task in problem gamblers, compared to healthy controls [31].

To date, impaired inhibition and enhanced salience attribution in substance dependent disorders has only been studied in separate designs, i.e. neutral Go/Nogo tasks in inhibition studies [22] and cue-reactivity tasks in salience attribution studies [7]–[11], [33]. Functional MRI studies examining the interaction between cognitive control (IFC, DLPFC, ACC) and salience attribution (amygdala, striatum, VLPFC) in substance dependent individuals or problem gamblers are currently lacking. We therefore employed a modified Go/Nogo task by including affective stimulus blocks (gambling, positive and negative), in addition to the standard affectively neutral block in problem gamblers (PRGs) and healthy controls (HCs). Subjects were requested to respond or withhold a response to specific types of pictures with a different affective loading, allowing the investigation of the interaction between motor inhibition and salience attribution.

Based on the attenuated BOLD response to affective stimuli in problem gamblers [13] and SUDs [34], [35], we hypothesized that PRGs would show a decreased BOLD response to positive and negative pictures compared to HCs in salience/motivational brain circuitry. Based on the findings of an enhanced neuronal response to gambling-related cues in PRGs [5], [6], we also hypothesized that PRGs compared to HCs would show enhanced brain activity during gambling related pictures in the salience/motivational circuitry (e.g. amygdala, striatum, VLPFC). Based on the I-RISA model, we hypothesized that compared to HCs, PRGs would show impaired response inhibition and diminished DLPFC, ACC and IFC activity in the context of neutral, positive and negative stimuli and even more so when confronted with an inhibition task in the context of gambling-related pictures compared to HCs.

## Methods

### Ethics Statement

The ethical review board of the Academic Medical Center approved the study and written informed consent was obtained from all subjects.

### Subjects

Sixteen problem gamblers (PRGs) and 15 healthy controls (HCs) participated in this study. PRGs were recruited from Dutch addiction treatment centres where they received cognitive behavioural therapy. HCs were recruited through advertisements in local newspapers. Because most treatment-seeking PRGs are men, only male participants were included in the study.

The main inclusion criterion for PRGs was current treatment for gambling problems. PRGs were interviewed with section T of the Diagnostic Interview Schedule [36] to assess the diagnostic criteria for a DSM-IV-TR diagnosis of pathological gambling. In addition, the South Oaks Gambling Screen (SOGS) [37] was administered, as a general indication of the severity of gambling problems and to facilitate comparisons with other studies using the SOGS.

Exclusion criteria for both groups were: lifetime diagnosis of schizophrenia or psychotic episodes; diagnosis of manic disorder (CIDI, section F), obsessive compulsive disorder (CIDI, section E), alcohol use disorders (CIDI, section J), substance dependent disorder (CIDI, section L) or post-traumatic stress disorder (CIDI, section K); treatment for mental disorders other than pathological gambling in the past 12 months; use of psychotropic medication; difficulty reading Dutch; age under 18 years; positive urine screen for alcohol, amphetamines, benzodiazepines, opioids or cocaine; history or current treatment for neurological disorders, major internal disorders, brain trauma, or exposure to neurotoxic factors. In addition, HCs were excluded if they gambled more than twice a year. To obtain a measure of subjects' global information processing speed, we administered the subscales Digit span and Number-Letter sequencing from the Wechsler Adult Intelligence Scale-Revised and combined these in a composite score for information processing speed (WAIS-R) [38].

Participants were reimbursed with 50 Euros transferred to their bank account following participation.

### Paradigm

In order to test inhibition in the context of neutral and affective pictures we designed a Go/Nogo task that consisted of four blocks containing pictures that were positive, negative, neutral, or gambling-related. The positive, negative, and neutral pictures were selected from the International Affective Picture System (IAPS) [39] based on their valence and arousal scores. While positive pictures _(mean: 7.6, SD 1.5)_ were higher in valence than neutral _(mean: 5.3, SD 3.5)_ and negative pictures _(mean: 2.4, SD 1.5)_, there were no differences in arousal scores between the positive and negative pictures _(positive mean: 5.6, SD 2.1, negative mean: 5.2, SD 2.2, neutral mean: 3.5, SD 2.0)_ (Lang et al. 2008). Gambling related pictures were taken from casino scenes, previously used in a study by Goudriaan et al. [5]. Pictures in each block were matched on visual properties such as brightness and complexity.

**Figure 1:**
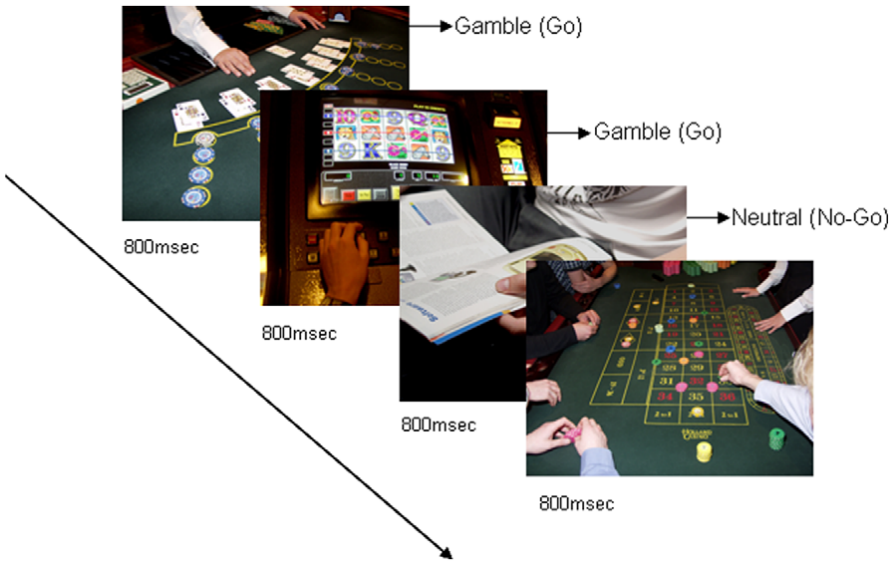
Example of the Go/Nogo Gamble block. Participants had to respond to gambling related pictures and try to withhold a response to neutral pictures.

Before each block started, an instruction appeared on the screen for 15 seconds, instructing participants to press a button when a certain type of stimulus was shown (Go trials) and to inhibit pressing the button when a neutral stimulus type was shown (No-Go trials). Each block consisted of 35 pictures, which were shown 4 times, presented in rapid succession for 800 ms each. To evoke an automated response, 100 Go trials and 40 No-Go trials were randomly presented. No-Go trials never occurred more than twice in a row. In the gambling block, for example, the instruction was to respond as accurately and fast as possible to gambling-related pictures, and not to respond to neutral pictures (see Figure 1). Because all pictures were neutral in the neutral block, participants were instructed to respond to all neutral pictures, but not to respond when a vehicle was shown in the picture (40 of the 140 trials).

Behavioural outcomes of interest included percentage of impulsive errors (responding to No-Go trials) and mean reaction times in the different blocks. Additional post hoc analyses were performed to test the signal detection accuracy and speed-accuracy trade-off between groups (please see Data S1).

### Procedure

An 8-item gambling urge questionnaire, with a range of 1–7 [40] was included to assess the degree of gambling craving. All subjects completed the urge questionnaire before and immediately after the gamble block during fMRI scanning.

Outside the scanner, subjects were trained on the Go/Nogo task. The practice session contained 90 pictures (70 Go pictures and 20 No-Go pictures) per block to ensure that participants were familiar with the task demands during each block and the assignment of the response button. In addition, participants were trained to use the response buttons to answer the gambling urge questions, to ensure that craving ratings could be obtained while in the scanner.

### Imaging Acquisition and Pre-Processing

Imaging data were obtained using a 3.0 Tesla Intera MRI scanner (Philips Medical Systems, Best, The Netherlands) with a phased array SENSE RF eight-channel receiver head coil. A total of 35 axial slices (voxel size 2.29×2.29×3 mm), no interslice gap, matrix size 96×96 mm, TR/TE = 2.3 s/30 ms, bandwidth 90 kHz) of T2*-weighted echo planar images (EPIs) sensitive to blood oxygenation level-dependent (BOLD) contrast were obtained, covering the entire brain except for the inferior regions of the cerebellum. A T1-weighed structural scan was made for co-registration with the fMRI data (voxel size 1×1×1 mm; 170 slices). Imaging analysis was performed using SPM5 (Statistical Parametric Mapping; Wellcome Trust Centre for Neuroimaging, London, UK). Images were manually reoriented and subsequently slice-timed, realigned and unwarped. Next, images were warped to MNI space using each subject's co-registered T1 image, and spatially smoothed using an 8 mm FWHM Gaussian kernel.

### Statistical Analysis

Individual mean reaction times were based solely on correct responses. All analyses were performed using SPSS 16 [41]. Demographical were analyzed using univariate analysis of variance (ANOVA). Reaction time data were tested for differences between groups, conditions and group×condition interactions with repeated measures ANOVA with conditions as within subject effects. This was followed up by separate ANOVA analyses to test group differences on the separate conditions. Non-normally distributed data (i.e. SOGS, craving scores, percentage of errors) were analyzed using Mann-Whitney U-tests for the comparison between groups. Friedman's ANOVAs were used to test differences between experimental conditions within groups (for the craving scores and percentage of errors during the various blocks) followed up by Wilcoxon tests for post-hoc comparisons. Because of significant reaction time differences between groups we performed additional Spearman correlation analyses to test for the relationship between RT and percentage of impulsive errors. All analyses were performed two-tailed with an alpha of 0.05. Furthermore, post-hoc analyses were conducted to test whether there were significant differences in detection of *signal to noise ratio* (correct responses – false alarms). For visual display purposes only, *speed-accuracy trade-off* (i.e inverse efficiency) scores were calculated. Please see the Data S1 section for details on methods and results.

All fMRI data were analysed within the context of the General Linear Model, using delta functions convolved with a canonical hemodynamic response function to model responses to each type of stimulus that was correctly responded to [(affective block×Go/NoGo) resulting in 8 regressors]. Incorrect responses were also included as a regressor in the design matrix but were not used in the fMRI analysis because there were not enough incorrect responses to have sufficient power to analyse them.

Contrast images containing parameter estimates were entered into a second-level (random effects) analysis.

Group interactions were investigated using specific a-priori regions of interest (ROIs) with a threshold set at p<.05, Family Wise Error (FWE) corrected for multiple comparisons across the search volume of 10 mm centred around a peak activation [small volume correction (SVC)] [42], [43]. We defined DLPFC, IFC, and ACC as a-priori ROIs given their role in response inhibition [16]–[19] and amygdala, ventral striatum, and VLPFC as a-priori ROIs in view of their involvement in salience attribution and cue reactivity [5], [6], [13], [15]. All ROIs were defined using the WFU PickAtlas Tool v2.4 [44] that incorporates the automatic anatomical labelling (AAL) atlas [45]. The templates of the superior frontal cortex and superior medial prefrontal cortex were used to assess activity in the DLPFC. Activity in the VLPFC was detected by using the templates of the middle orbitofrontal cortex and inferior orbitofrontal cortex.

To test the effect of *salience attribution* we investigated the contrasts: Gamble Go – Neutral Go, Positive Go – Neutral Go, and Negative Go – Neutral Go. *Response inhibition* was investigated with the contrast: Neutral NoGo – Neutral Go. Finally, and most importantly, the *interaction between salience and response inhibition* was examined by comparing the response inhibition activations in the different affective/salience conditions with the contrasts:, Gamble NoGo - Neutral NoGo, Positive NoGo – Neutral NoGo, and Negative NoGo - Neutral NoGo.

## Results

### Sample characteristics

Table 1 summarizes demographic and clinical characteristics for PRGs and HCs. There was no significant difference between the groups in terms of age, and general cognitive performance (composite score on the subscales Digit Span and Number-Letter sequencing from the WAIS-R). As expected, PRGs had significantly higher SOGS scores than HCs and all PRGs fulfilled criteria for ‘probable pathological gambler’ defined by a SOGS score of five or more. Furthermore, except for one PRG who met 4 criteria for PG instead of 5 criteria, all PRGs met criteria of a current DSM-IV-TR pathological gambling diagnosis.

**Table 1:** Demographic and clinical information of participants.

	HCs N = 15	PRGs N = 16	Significance (ANCOVA; Mann-Whitney U)
Age, mean (SE)	36.20 (10.69)	34.38 (11.14)	*F*(1,30) = 0.221 *p* = 0.65
WAIS composite score, mean (SE)	15.40 (1.02)	13.75 (0.71)	*F*(1,30) = 1.804 *p* = 0.19
SOGS*, mean (SE)	0.07 (0.26)	11.57 (3.00)	U = 0, p = 0.000
Gambling craving before task*, mean (SE)	8.27 (2.58)	16.56 (10.26)	U = 50, p = 0.005
Gambling craving after task, mean, (SE)	17.80 (13.06)	21.50 (11.63)	U = 87, p = 0.202

HCs = Healthy controls, PRGs = Problematic gamblers, WAIS composite score = composite score of the subscales Digit span and Number-Letter sequencing from Wechsler Adult Intelligence Scale-Revised; SOGS = South Oaks Gambling Screen, SE = standard error;

* = significant group difference at p<0.05.

Before scanning, PRGs had a significantly higher average gambling craving score than HCs (see Table 1). However, after performing the gamble block, gambling craving scores were increased in both groups (for HCs: (χ^2^(1) = 8.07, p<0.005; and for PRGs: (χ^2^(1) = 4.57, p<0.033), and there was no group difference on gambling craving after the gamble block (see Table 1).

### Behavioural performance on the Go/Nogo task

**Figure 2:**
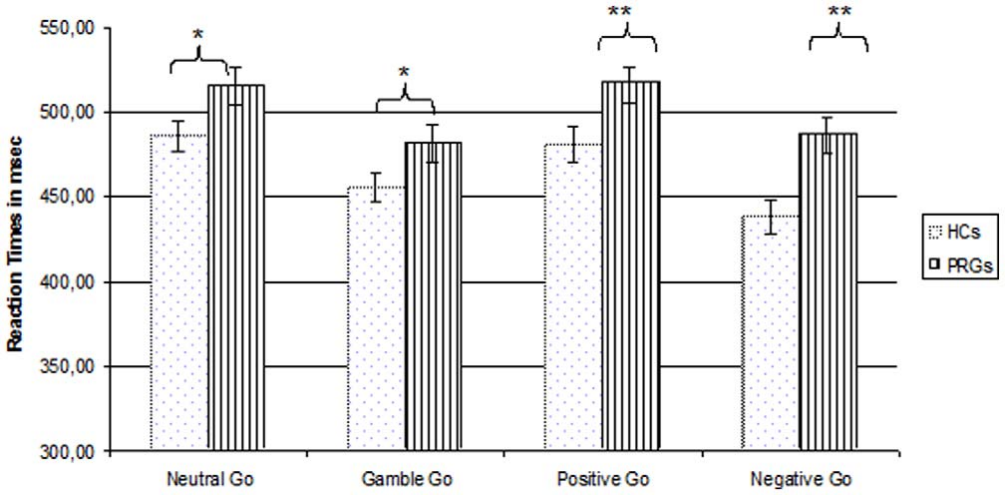
A: Reaction time during the different Go/NoGo blocks. HCs = Healthy controls, PRGs = problematic gamblers, msec = milliseconds; ** = significant group difference at p<0.05; * = trend for group differences *p*<0.10; Error bars represent the standard errors of the mean. B: Percentage of impulsive errors during Go/NoGo blocks. HCs = Healthy controls, PRGs = Problematic gamblers, ** = significant group difference at p<0.05; * = trend for group differences *p*<0.10; Error bars represent the standard deviations of the mean.

Behavioural data for one HC was lost. Therefore, 15 instead of 16 HCs were used for the behavioural analyses. Overall, there was a significant main effect for condition _(F(3,26) = 22.059, p = 0.001)_ and for group _(F(1,29) = 8.075, p = 0.008)_. PRGs responded slower than HCs _(PRGs Mean = 500.36 msec, SE = 8.61 and HCs Mean = 465.19 msec, SE = 8.89)_. PRGs were significantly slower compared to HCs during the negative stimulus block _(PRGs Mean = 487.04, SE = 10.05 and HCs Mean = 438.32, SE = 10.38 ; F(1,30) = 11,363, p = 0.002)_ and during the positive block _(PRGs: Mean = 517.10, SE = 9.97; HCs: Mean = 480.78 SE = 10.29; F(1,30) = 6.429, *p* = 0.017)_, whereas a trend was present for the neutral block _(PRGs: Mean = 515.58, SE = 10.37; HCs: Mean = 486.15, SE = 10.71; F(1,30) = 3.899, *p* = 0.058)_ and for the gamble block _(PRGs: Mean = 481.70, SE = 9.49; HCs: Mean = 455.52, SE = 9.80; F(1,30) = 3.679, *p* = 0.065)_ (see Figure 2A). However, PRGs made significantly less impulsive errors compared to HCs during the gambling block _(PRGs: Mean = 7.97, SD = 6,91; HCs: Mean = 17.67, SD = 8.63; U = 41.050, p = 0.001)_ and a trend in the same direction was present in the positive block _(PRGs: Mean = 13.28, SD = 8.15; HCs: Mean = 21.00, SD = 13.02; U = 73.50, p = 0.066)_ (see Figure 2B).

A within-group repeated measures analysis showed a significant effect of stimulus condition on the percentage of impulsive errors in the HCs _(χ2(3) = 8.69, p<0.034)_. Post-hoc analyses indicated that HCs performed best during the negative block compared to the other blocks _(negative block compared to neutral block: T = 5, p<0.007, negative block compared to gamble block: T = 231, p<0.034, negative block compared to positive block: T = 7.5, p<0.008)_. Also in PRGs, a significant effect of stimulus condition on the percentage of impulsive errors was present _(χ2(3) = 17.34, p<0.001)_. Here, post-hoc tests showed that PRGs performed best during the gamble block compared to the other blocks _(gamble - neutral block: T = 6.5 p<0.001, gamble - positive block: T = 23.5 p<0.038, gamble - negative block: T = 9.5 p<0.020)_. Furthermore, PRGs made fewer impulsive errors during the positive and negative block compared to the neutral block _(positive block compared to neutral block: T = 25, p<0.046, negative block compared to neutral block: T = 11, p<0.005)_. There was no performance difference between the positive and negative block in PRGs.

Results from the Spearman correlation analyses showed only one significant negative correlation, between the percentage of impulsive errors on the positive condition and reaction time (r = −0.379, N = 30, p = 0.030), indicating that in the positive condition, slower response times were associated with better task performance across groups. However, when testing the Spearman correlations in each group separately we found no significant correlations between the percentage of impulsive errors and reaction times.

### fMRI results

#### Salience attribution

To test differences in salience attribution towards affective stimuli in groups, we compared brain activation during gambling Go pictures and non-monetary positive and negative Go pictures to brain activation during neutral Go pictures. For the main effects of these contrasts, we refer the reader to the Data S1. Here we only present group interactions regarding the salience of the different stimuli.

### Gambling pictures

#### Group interaction Gamble Go versus Neutral Go

**Figure 3:**
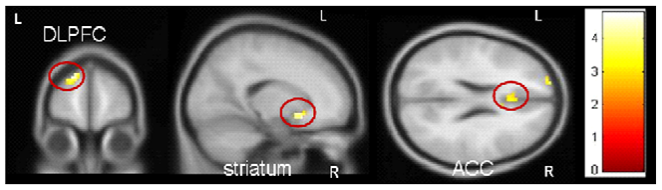
Group interaction gamble pictures – neutral pictures. PRGs showed more activation in the left dorsolateral prefrontal cortex, right ventral striatum, and right anterior cingulate, than HCs. Results are depicted with a threshold of p<0.001 uncorrected to show the extent of activation. Colour bar represents corresponding T values.

PRGs showed more activity in regions associated with salience attribution compared to HCs on Gamble Go vs. Neutral Go: left DLPFC (peak voxel: *x*, *y*, *z* = −15, 60, 30, *T* = 4.46, *p*_FWE_ = 0.003), right ventral striatum (peak voxel: *x*, *y*, *z* = 15, 15, −9, *T* = 4.79, *p*_FWE_ = 0.001), and right ACC (peak voxel: *x*, *y*, *z* = 6, 21, 30, *T* = 4.45, *p*_FWE_ = 0.002) ( Figure 3). HCs showed no areas that were more active than in PRGs.

### Positive pictures

#### Group interaction Positive Go versus Neutral Go

**Figure 4:**
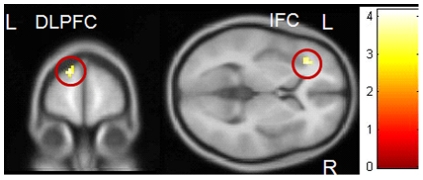
Group interaction positive pictures – neutral pictures. PRGs showed more activation in the left dorsolateral prefrontal cortex and left inferior frontal gyrus than HCs. Results are depicted with a threshold of p<0.001 uncorrected to show the extent of activation. Colour bar represents corresponding T values.

PRGs showed more activity compared to HCs while watching positive Go pictures vs. neutral Go pictures in left DLPFC (peak voxel: *x*, *y*, *z* = −15, 60, 30, *T* = 4.18, *p*_FWE_ = 0.006) and left IFC (peak voxel: *x*, *y*, *z* = −33, 33, 3, *T* = 4.01, *p*_FWE_ = 0.012). HCs showed no areas that were more active than in PRGs (Figure 4).

### Negative pictures

#### Group interaction Negative Go versus Neutral Go

**Figure 5:**
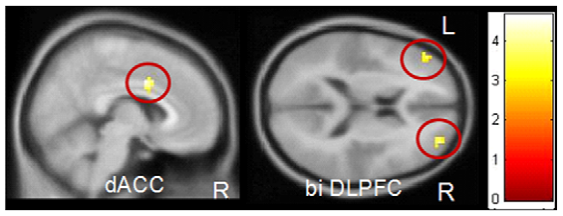
Group interaction negative pictures – neutral pictures. PRGs showed more activation in the bilateral dorsolateral prefrontal cortex and right dorsal cingulate cortex than HCs. Results are depicted with a threshold of p<0.001 uncorrected to show the extent of the activation. Colour bar represents corresponding T values.

PRGs showed more activity compared to HCs on the Negative Go pictures vs. Neutral Go pictures in right dorsal cingulate cortex (peak voxel: *x*, *y*, *z* = 6, 3, 36, *T* = 4.66, *p*_FWE_ = 0.003) and bilateral DLPFC (peak voxel: *x*, *y*, *z* = 33, 54, 15, *T* = 4.11, *p*_FWE_ = 0.011 and peak voxel: *x*, *y*, *z* = −45, 42, 15, *T* = 3.63, *p*_FWE_ = 0.029 ). HCs revealed no regions that were more active than in PRGs (see Figure 5).

### Neutral Response Inhibition

#### Group interaction Neutral NoGo versus Neutral Go

**Figure 6:**
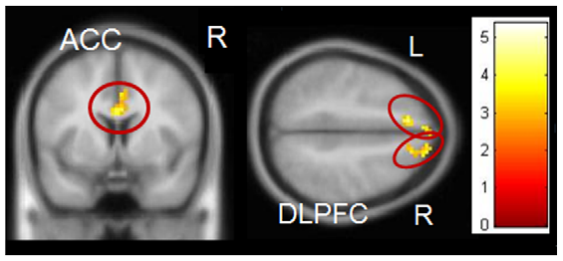
Group interaction neutral inhibition. PRGs showed more activation in the bilateral dorsolateral prefrontal cortex cortex, and right anterior cingulate than HCs. Results are depicted at a threshold of p<0.001 uncorrected. Colour bar represents the corresponding T values.

PRGs activated more areas associated with response inhibition and conflict monitoring than HCs during neutral inhibition: bilateral DLPFC (peak voxel: *x*, *y*, *z* = 12, 45, 51, *T* = 4.82, *p*_FWE_ = 0.001 and peak voxel: *x*, *y*, *z* = −9, 30, 51, *T* = 5.31, *p*_FWE_ = 0.001) and right ACC (peak voxel: *x*, *y*, *z* = 1, 6, 27, *T* = 4.13, *p*_FWE_ = 0.011), see Figure 6). HCs showed no regions that were more activated than PRGs during neutral inhibition.

### Response inhibition during affective blocks

The effect of affective stimuli on response inhibition was investigated by analysing the BOLD response during No-Go trials in the affective block vs. No-Go trials in the neutral block. For main effects in the groups, the reader is referred to the Data S1. Here we only present group interactions regarding the effect of affective stimuli on response inhibition.

### Inhibition during gamble pictures

#### Group interaction Gamble NoGo versus Neutral NoGo

**Figure 7:**
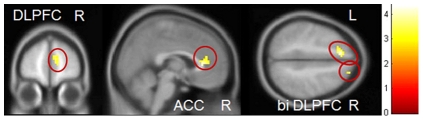
Group interaction inhibition during gamble pictures. HCs showed more activation in the bilateral dorsolateral prefrontal cortex, right ventral lateral prefrontal cortex, and right anterior cingulate than PRGs. Results are depicted with a threshold of p<0.001 uncorrected. Colour bar represents corresponding T values.

PRGs showed no regions that were more activated than in HCs during gamble compared to neutral No-Go trials. HCs showed more bilateral DLPFC (peak voxel: *x*, *y*, *z* = 21, 42, 45, *T* = 3.60, *p*_FWE_ = 0.034 and peak voxel: *x*, *y*, *z* = −12, 27, 48, *T* = 4.41, *p*_FWE_ = 0.005), right DLPFC (peak voxel: *x*, *y*, *z* = 9, 54, 15, *T* = 3.68, *p*_FWE_ = 0.028), and right ACC (peak voxel: *x*, *y*, *z* = 3, 30, 9, *T* = 4.05, *p*_FWE_ = 0.011) activity than PRGs during gamble No-Go trials compared to neutral No-Go trials (See Figure 7).

### Inhibition during positive pictures

#### Group interaction Postive NoGo versus Neutral NoGo

**Figure 8:**
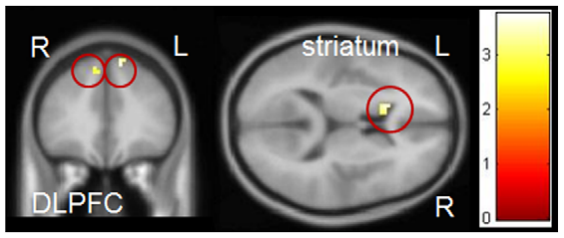
Group interaction during positive pictures. HCs showed more activation in the bilateral dorsolateral prefrontal cortex, and left ventral striatum than PRGs. Results are depicted with a threshold of p<0.001 uncorrected. Colour bar represents corresponding T values.

There were no regions that were more activated in PRGs compared to HCs during positive inhibition. HCs showed increased activation in bilateral DLPFC (peak voxel: *x*, *y*, *z* = 12, 33, 54, *T* = 3.74, *p*_FWE_ = 0.045 and peak voxel: *x*, *y*, *z* = −9, 30, 51, *T* = 3.77, *p*_FWE_ = 0.025) and left ventral striatum peak voxel: *x*, *y*, *z* = −15, 18, 21, *T* = 3.75, *p*_FWE_ = 0.026) compared to PRGs during positive inhibition (see Figure 8).

### Inhibition during negative pictures

#### Group interaction Negative NoGo versus Neutral NoGo

**Figure 9:**
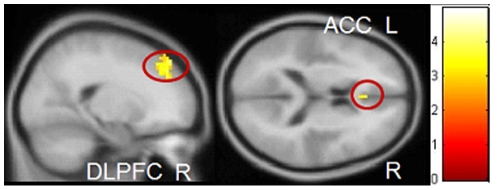
Group interaction inhibition during negative pictures. HCs showed more activation than PRGs in the right dorsolateral prefrontal cortex, and left anterior cingulate. Results are depicted with a threshold of p<0.001 uncorrected. Colour bar represents corresponding T values.

PRGs showed no regions that were more activated than HCs in negative compared to neutral No-Go trials. HCs activated the right DLPFC (peak voxel: *x*, *y*, *z* = 24, 42, 45, *T* = 4.95, *p*_FWE_ = 0.001) and left ACC (peak voxel: *x*, *y*, *z* = −6, 51, 0, *T* = 3.87, *p*_FWE_ = 0.011) more than PRGs during negative No-Go compared to neutral No-Go trials (see Figure 9).

## Discussion

### Problem gamblers show enhanced salience to gambling and positive pictures

Congruent with our hypothesis regarding salience attribution towards gambling pictures in PRGs, we found that PRGs who were confronted with gambling related cues versus neutral pictures showed increased DLPFC, ACC and ventral striatum activation compared to HCs. This is in line with previous findings on cue reactivity in PRGs, showing enhanced DLPFC and ACC activity in PRGs compared to HCs during passive gamble picture viewing [5], [6]. Interestingly, we also found enhanced ventral striatum activity congruent with findings of cue reactivity studies in substance dependent subjects, implicating the involvement of the reward system in cue reactivity in PRGs as well [46]–[48]. In addition, we hypothesized that problem gamblers would be less sensitive to positive and negative pictures, which would be reflected in attenuated responses in the brain circuitry involved in salience and motivation processing (amygdala, striatum, and VLPFC). Unexpectedly, we found increased activation in the DLPFC and the IFC in PRGs compared to HCs when comparing positive with neutral pictures. Similarly, negative pictures elicited more DLPFC and dorsal cingulate activity in PRGs compared to HCs. These areas are associated with error monitoring and risk perception [49], [50]. These findings are at odds with findings in SUDs, where attenuated responses to monetary wins and losses, and to positive pictures, have been repeatedly reported [7], [10], [11], [34], [35], although an increased response to positive pictures in alcohol dependent subjects has also been found [51]. These discrepant findings may be explained by methodological factors, in particular stimulus duration and associated cognitive demand.

### During neutral inhibition, PRGs perform similar to HCs, but PRGs recruit additional brain regions

Contrary to our hypothesis of impaired response inhibition in combination with *diminished* DLPFC, ACC and IFC activity during the neutral block in PRGs compared to healthy controls, we found *increased* DLPFC and ACC activity in PRGs compared to HCs during neutral inhibition, whereas accuracy was similar between PRGs and HCs. However, PRGs tended to have longer reaction times compared to HCs. Together; these findings suggest a more effortful strategy in PRGs to perform at a similar level as HCs. Our findings are congruent with neuroimaging studies in substance dependent populations that reported enhanced regional brain activity in SUD groups compared to HCs in the absence of performance differences [23], [24].

### During gamble and positive blocks, PRGs perform better than HCs

Our study is the first to test the hypothesis of decreased inhibition in PRGs compared to HCs when confronted with non-monetary affective and with gamble related cues. Surprisingly, we did not find behavioural evidence of decreased inhibitory control in PRGs during affective Go/NoGo blocks. On the contrary, PRGs made less commission errors during inhibition trials in the gambling and positive blocks than HCs. This effect cannot be solely explained by the longer reaction times of PRGs, because we did not find significant correlations between reaction times and impulsive errors, except for an overall correlation in the positive condition, across groups. When studying the *within-group* differences between conditions we found that in PRGs performance during gamble, positive and negative pictures was better than during neutral pictures. Notably, in PRGs, performance during the gamble block was most accurate compared to the other blocks. Within-group differences in HCs only showed a better performance on negative pictures compared to all other blocks. These behavioural findings can be interpreted as evidence that subjects perform best when confronted with group-specific relevant stimuli (i.e., gamble, positive and negative pictures for PRGs and negative pictures only for HCs).

Although our fMRI results showed that during neutral inhibition PRGs recruited more areas associated with cognitive control than HCs, PRGs showed less cognitive control activity during gambling inhibition trials compared to neutral inhibition trials as indicated by attenuated activation of DLPFC, and ACC compared to HCs. Similarly, during positive inhibition PRGs showed lower DLPFC and ventral striatum activation compared to HCs, whereas PRGs also showed lower activation of DLPFC and ACC during negative inhibition compared to HCs. The observation that PRGs recruit fewer areas associated with cognitive control (DLPFC, ACC) and affective processing (ventral striatum) during response inhibition in affective conditions compared to HCs, while performing better than HCs during gamble and positive conditions, suggests that gambling and positive pictures facilitate task performance in PRGs, whereas this is not the case for HCs. As an alternative, although not mutually exclusive, interpretation we suggest that HCs experience more interference from positive and gamble stimuli compared to PRGs: *within* group comparisons in HCs showed more activation of cognitive control areas (such as IFC and ACC) during affective versus neutral No-Go trials, differences which were absent in PRGs (see Data S1). Thus, HCs may need to increase prefrontal recruitment to perform adequately during positive and gamble conditions, contrary to PRGs.

The fact that emotionally salient stimuli can capture attention and influence task performance is a consistent finding in a variety of paradigms [52]–[54]. The “dual process and competition” framework describes the interaction between motivational and cognitive functioning and suggests that affective stimuli influence competition both at the perceptual and executive level [53]. For example, when affective stimuli are salient for the person, the spatial locus of the stimuli attracts extra attention, facilitating certain task performances, such as discrimination or response inhibition tasks. However, affective stimuli may also prove to be overwhelming, resulting in an overload of attentional resources towards these affective stimuli, resulting in deficient cognitive control [53]. Our findings of gamble related and positive pictures facilitating task performance more in PRGs than HCs may therefore indicate that these stimuli are more (but not overwhelmingly) relevant to PRGs than for HCs.

Enhanced attention for addiction related cues, i.e. attentional bias, is a key cognitive process related to cue reactivity and involves the tendency of addicted individuals to automatically allocate and maintain increased attention to addiction related cues [54], [55]. Attentional bias can result in impaired cognitive task performance, as in addiction-Stroop tasks in which enhanced attention towards addiction words distracts the addicted person from the actual task [56], [57]. However, attentional bias can also *enhance* performance in addicted persons, as in dot-probe paradigms in which detecting probes behind addiction related pictures is facilitated in comparison to neutral pictures [58]. A recent study in problem gamblers using an attentional blink paradigm in problem gamblers indicated an enhanced ability to process gambling-related information compared to controls [59]. The *incentive sensitization* theory states that attentional bias develops due to repeated exposure to addiction related stimuli, which results in sensitization of the mesocorticolimbic system to such stimuli (Robinson and Berridge, [60]. Our finding of cue-reactivity towards gamble pictures in PRGs compared to HCs is indeed in line with incentive sensitization; attentional bias for gamble related stimuli, associated with an upregulated mesolimbic response towards these gamble pictures could thus arguably have resulted in better performance during the gambling block in PRGs compared to HCs.

PRGs also tended to perform more accurately than HCs during positive picture viewing, again indicating facilitation of appropriate responding due to increased attention and motivation towards positive affective pictures in PRGs compared to HCs [61]–[63]. This explanation is supported by our finding that positive pictures elicited more activation in regions associated with salience coding and cognitive control (i.e., VLPFC and inferior frontal cortex) in PRGs compared to HCs. However, additional research is needed to investigate why positive pictures are more salient for PRGs than for HCs. An alternative explanation for our findings could be that the enhanced activity in PRGs compared to HCs during affective Go trials relative to neutral Go trials reflects greater tonic control activity during the affective conditions, so that PRGs do not have to increase recruitment of cognitive control areas during NoGo trials to the same extent as HCs.

Finally, during the negative affective block, task performance was also facilitated compared to neutral pictures, in both HCs and PRGs. The fact that negative pictures can facilitate attention and increase task performance compared to neutral pictures has been consistently found in various tasks [64], [65]. This finding is intuitively sensible; an organism should be alert to potential threatening cues for its survival and thus must allocate more attention to a task when negative (threatening) stimuli are presented.

### Strengths and limitations, and suggestions for future research

This is the first study in problem gambling testing the effect of affective stimuli on response inhibition, and has both strengths and limitations. Strengths include the use of a paradigm that probes motivational as well as cognitive systems simultaneously, providing the opportunity to study their interaction in problem gamblers.

A limitation is that we did not assess subjective valence or salience ratings of the pictures by the participants themselves. Therefore, we can only infer that the enhanced activity in mesolimbic areas during positive picture watching reflects a higher salience of these pictures for PRGs. However, we did select our pictures based on the IAPS valence and arousal ratings, which are well validated and tested on an extensive number of people [39]. Furthermore, we did not incorporate measures of arousal which is likely to be relevant during processing of affective stimuli in healthy controls [66]–[68]. Arousal induced by affective pictures could have had a differential effect on PRGs compared to HCs. For example, it has been suggested that positively reinforcing properties of arousal during gambling may be more important than actual monetary gains in the maintenance of gambling behavior [69]. Hence, excitement may represent ‘the gambler's drug’ [70]. Furthermore, in pathological gamblers dopamine release in the ventral striatum appears to be associated with increased excitement levels [71]. Thus, future research could benefit from including measures of physiological arousal in addition to subjective ratings of arousal, to further understand the influence of affective pictures on behavioral inhibition and its neural correlates in PRGs.

Another difficulty is the fact that we found significant reaction time differences between groups on all conditions. Controlling for RT differences between groups is an important, albeit somewhat controversial, issue in fMRI studies. Whereas it has been advised to include RTs in first-level (single-subject) models when these exceed 2–3 s, short events (RT<1 s) are routinely modelled using delta functions due to the sluggishness of the haemodynamic response. Adding RTs in these rapid event-related designs will introduce a scaling factor which may confound interpretation of regional effects, which is why in the present study, we chose to analyse our fMRI data in a straightforward manner. However, actual time spent on the task could have influenced our observed activations [70], and this should be kept in mind when interpreting these results.

The fact that our affective blocks were not presented in counterbalanced order could have introduced potential confounds due to practice effects or fatigue. However, as shown by our behavioural results, response inhibition errors did not diminish or increase over time. In addition, possible carry-over effects of cue reactivity during the gambling block to the other two blocks seem unlikely because there was a considerable amount of time between these blocks, during which a craving questionnaire was presented, followed by a 15 second presentation of instructions for the next condition.

Finally, as expected, we found higher levels of baseline craving in PRGs compared to HCs. Surprisingly, this group difference disappeared after watching the gambling pictures. A possible explanation is that the craving questionnaires involved questions on whether the person would accept to gamble if given the opportunity, or whether the person thought that he or she would enjoy gambling in that instance. Given the fact that the HCs did not experience any problems with gambling at baseline, and that gambling is an attractive entertainment for most people, the higher scores on the craving questionnaires in HCs after viewing gambling games are likely to reflect something different (an interesting option) than the craving reported by PRGs with their history of gambling problems (an irresistible urge).

Our findings are clinically relevant because this study shows that PRGs rely on compensatory brain activity coupled with slower response times to perform similar to HCs on a neutral response inhibition task, but that salient affective stimuli facilitate response inhibition in PRGs. Prefrontal cortex functioning, crucial for executive functions such as response inhibition, is modulated by ascending projections of e.g. noradrenalinergic and dopaminergic neurons [72]. A dysfunctional dopamine system has adverse effects on cortical-striatal loops and is associated with compromised prefrontal cortex functioning [for a review 73]. Pathological gambling has been associated with lower dopamine receptor density [74]–[77] and in pathological gamblers decreased dopamine binding has been reported during gambling games compared to healthy controls [78]. Therefore, in our study, salient stimuli which are known to enhance DA transmission, especially in the reward system [79], [80], could have transiently restored the normally hypoactive dopaminergic state of PRGs and facilitated normal prefrontal functioning. However, numerous other neurotransmitter systems (e.g., serotonin, glutamate and opiates) are engaged in the processing of affective stimuli and may also affect prefrontal cortex functioning [81]. Furthermore, effects of modulatory neurotransmitter input to the prefrontal cortex are likely to be nonlinear, so that increasing levels of activity in the ascending monoaminergic systems result in an inverted U-shape function of behavioral performance [72]. Thus, whereas some prefrontal cortex functions could benefit from enhanced dopaminergic transmission, other functions could deteriorate. Our findings of enhanced BOLD responses in reward and motivation systems are not a direct measure of neurotransmission function and more research is therefore needed to understand the complex interaction between motivational functions and cognitive functions in PRGs. Future research could benefit from Positron Emission Tomography (PET) and single proton emission computed tomography (SPECT) studies, in which binding to dopamine receptors, dopamine transmission and dopamine receptor availability can be studied.

### Conclusion

This study shows that gambling-related and other affective stimuli are more salient for PRGs than for HCs. Also, compared to HCs, PRGs rely on compensatory brain activity to achieve similar performance during neutral response inhibition. A gambling-related or positive context, however, appears to facilitate response inhibition in PRGs as indicated by lower brain activity and fewer behavioural errors in PRGs compared to HCs. These findings indicate that certain motivational processes need not interfere with cognitive function but instead can enhance performance in PRGs.

## Supporting Information

Data S1 (DOC)
