# Supplementary material for: Response Inhibition during Cue Reactivity in Problem Gamblers: An fMRI Study
Source: PLoS One. 2012 Mar 30;7(3):e30909. doi: 10.1371/journal.pone.0030909 (PMC3316530; doi:10.1371/journal.pone.0030909)
Supplement: Data S1 — (DOC) [file pone.0030909.s001.doc]

**Supplementary data:**

**Methods**

*Signal to noise ratios* were calculated to assess the response specificity of both groups. This signal to noise ratio was calculated by subtracting the Z scores of correct Go responses with Z scores of responses to NoGo stimuli. d’= Z (correct Go) – Z (response to NoGo). A high signal to noise ratio indicates that the subject was able to distinguish Go responses from No-Go responses, whereas a low signal to noise ratio indicates an inability to distinguish Go responses from No-Go responses.

*Speed-accuracy trade*-*off*, i.e. inverse efficiency scores, involves computing a ratio of RT over proportion correct responses, for each observer and condition, c’= (RT correct response/ accuracy of responses). They are interpreted in the same way as correct RT, being in fact identical when accuracy is perfect, and growing proportionately with increases in errors.

**Results**

There were no significant group ((F(1,28))=2.863, p= 0.102) or condition effects (F(3,84)=0.697, p=0.557) for the signal to noise ratios of the two groups. There was however a trend for a group x condition interaction effect (F(3,84)=2.374, p=0.076). Univariate ANOVAs investigating the specific group x condition effect, showed a trend for PRGs having a worse signal to noise ratio compared to HCs during the negative condition (F(1,28)=3.157, p= 0.086).


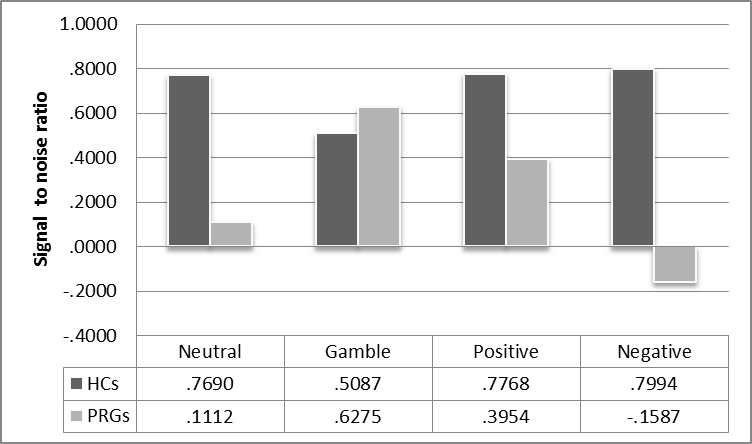


**Signal to noise ratios**: Numbers are mean signal to noise ratios per group per condition. A high signal to noise ratio indicates that the subject was able to distinguish Go responses from No-Go responses. HCs: healthy controls; PRGs: problem gamblers.

However, these speed-accuracy trade-off analyses should only be used when RTs are linearly correlated with task performance. Thus in our case, the speed-accuracy trade-off data could not be used for statistical analyses (Townsend and Ashby 1978,1983).


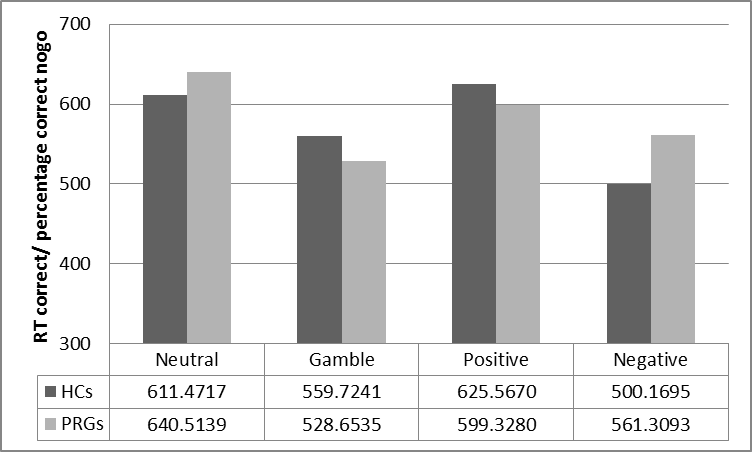


**Speed-accuracy trade-offs:** Numbers are mean speed-accuracy trade-off per group per condition in milliseconds. Vertical axis indicates time needed to perform accurately. HCs: healthy controls; PRGs: problem gamblers.

**fMRI results**

To test differences in salience attribution towards different affective stimuli in groups, we compared the BOLD response during affective Go pictures to the BOLD response during neutral Go pictures. Main group effects of each contrast were analyzed using one-way ANOVA implemented in SPM5, and are reported at p <0.001 uncorrected with an extent threshold of 10 voxels.

| **Healthy controls** | | | | | | **Pathological gamblers** | | | | |
| --- | --- | --- | --- | --- | --- | --- | --- | --- | --- | --- |
|  |  | **MNI coordinates** | | |  |  | **MNI coordinates** | | |  |
|  | **L/R** | **x** | **y** | **z** | **T value** | **L/R** | **x** | **y** | **z** | **T value** |
| **Prefrontal cortex** |  |  |  |  |  |  |  |  |  |  |
| Superior |  |  |  |  |  | R  L | -15  6 | 60  63 | 33  0 | 5.04  3.81 |
|  |  |  |  |  |  |
| Anterior cingulate |  |  |  |  |  | R | 0 | 45 | 3 | 3.61 |
| **Limbic lobe** |  |  |  |  |  |  |  |  |  |  |
| Precunues |  |  |  |  |  | R | 3 | -51 | 21 | 5.78 |
| **Parietal lobe** |  |  |  |  |  |  |  |  |  |  |
| Angular gyrus |  |  |  |  |  | R  L | 60  -51 | -54  -63 | 9  33 | 3.42  5.66 |

**Gamble Go > Neutral Go main effect per group.** BOLD responses for main effects are reported at p<0.001 with an extent threshold of 10 contiguous voxels; BOLD= blood oxygenation level-dependent; MNI= Montreal Neurological Institute

| **Healthy controls** | | | | | | **Pathological gamblers** | | | | |
| --- | --- | --- | --- | --- | --- | --- | --- | --- | --- | --- |
|  |  | **MNI coordinates** | | |  |  | **MNI coordinates** | | |  |
|  | **L/R** | **x** | **y** | **z** | **T value** | **L/R** | **x** | **y** | **z** | **T value** |
| **Prefrontal cortex** |  |  |  |  |  |  |  |  |  |  |
| Inferior |  |  |  |  |  | L | -51 | 24 | 12 | 5.05 |
| Medial Superior |  |  |  |  |  | R | 6 | 51 | 36 | 3.69 |
| Superior |  |  |  |  |  | L | -15 | 60 | 30 | 5.64 |
| Posterior cingulate |  |  |  |  |  | R | 3 | -12 | 39 | 5.13 |
| Anterior cingulate |  |  |  |  |  | R | 3 | 45 | 6 | 3.98 |
| **Temporal lobe** |  |  |  |  |  |  |  |  |  |  |
| Superior | R  L | 45  -51 | 30  45 | 0  3 | 4.20  4.42 |  |  |  |  |  |
|  |  |  |  |  |
| Middle | R  L | 48  -54 | -57  -63 | 12  12 | 4.36  4.34 | R  L | 54  -54 | -57  -63 | 15  12 | 4.98  6.06 |

**Positive Go > Neutral Go main effect per group.** BOLD responses for main effects are reported p<0.001 with an extent threshold of 10 contiguous voxels; BOLD= blood oxygenation level-dependent; MNI= Montreal Neurological Institute

| **Healthy controls** | | | | | | **Pathological gamblers** | | | | |
| --- | --- | --- | --- | --- | --- | --- | --- | --- | --- | --- |
|  |  | **MNI coordinates** | | |  |  | **MNI coordinates** | | |  |
|  | **L/R** | **x** | **y** | **z** | **T value** | **L/R** | **x** | **y** | **z** | **T value** |
| **Prefrontal cortex** |  |  |  |  |  |  |  |  |  |  |
| Medial superior |  |  |  |  |  | R  L | 6  -27 | 63  48 | 15  12 | 4.71  4.95 |
| Middle | L | -21 | 27 | 36 | 4.11 |  |  |  |  |  |
| Inferior frontal |  |  |  |  |  | R | 51 | 30 | 6 | 4.08 |
| Post central | L | -42 | -9 | 33 | 4.37 |  |  |  |  |  |
| **Limbic lobe** |  |  |  |  |  |  |  |  |  |  |
| Caudate | R | 9 | 5 | 16 | 3.46 |  |  |  |  |  |
| Precuneus |  |  |  |  |  | L | -3 | -51 | 15 | 4.50 |
| **Temporal lobe** |  |  |  |  |  |  |  |  |  |  |
| Middle | R  L | 57  -51 | -66  -69 | 3  3 | 4.55  4.62 | R  L | 57  -54 | -66  -63 | 3  12 | 3.83  5.44 |

**Negative Go > Neutral Go main effect per group.** BOLD responses for main effects are reported p<0.001 with an extent threshold of 10 contiguous voxels; BOLD= blood oxygenation level-dependent; MNI= Montreal Neurological Institute

| **Healthy controls** | | | | | | **Pathological gamblers** | | | | |
| --- | --- | --- | --- | --- | --- | --- | --- | --- | --- | --- |
|  |  | **MNI coordinates** | | |  |  | **MNI coordinates** | | |  |
|  | **L/R** | **x** | **y** | **z** | **T value** | **L/R** | **x** | **y** | **z** | **T value** |
| **Prefrontal cortex** |  |  |  |  |  |  |  |  |  |  |
| Superior |  |  |  |  |  | R | 12 | 45 | 51 | 5.48 |
| Anterior cingulate |  |  |  |  |  | R | 12 | 42 | 21 | 4.54 |
| **Limbic lobe** |  |  |  |  |  |  |  |  |  |  |
| Precuneus |  |  |  |  |  | R | 3 | -51 | 18 | 3.86 |
| **Temporal lobe** |  |  |  |  |  |  |  |  |  |  |
| Middle | R | 48 | -36 | -3 | 5.37 |  |  |  |  |  |
| **Parietal lobe** |  |  |  |  |  |  |  |  |  |  |
| Angular gyrus |  |  |  |  |  | R  L | 48  -48 | -69  -63 | 39  42 | 4.35  4.21 |

**Neutral NoGo > Neutral Go main effect per group.** BOLD responses for main effects are reported p<0.001 with an extent threshold of 10 contiguous voxels; BOLD= blood oxygenation level-dependent; MNI= Montreal Neurological Institute

To test differences in the effect of affective stimuli on response inhibition in groups, we compared the BOLD response during affective NoGo pictures to the BOLD response during neutral NoGo pictures. Main group effects of each contrast were analyzed using one-way ANOVA implemented in SPM5, and are reported at p <0.001 uncorrected with an extent threshold of 10 voxels.

| **Healthy controls** | | | | | | **Pathological gamblers** | | | | |
| --- | --- | --- | --- | --- | --- | --- | --- | --- | --- | --- |
|  |  | **MNI coordinates** | | |  |  | **MNI coordinates** | | |  |
|  | **L/R** | **x** | **y** | **z** | **T value** | **L/R** | **x** | **y** | **z** | **T value** |
| **Prefrontal cortex** |  |  |  |  |  |  |  |  |  |  |
| Anterior cingulate | R | 3 | 30 | 9 | 3.90 |  |  |  |  |  |

**Gamble NoGo > Neutral NoGo main effect per group.** BOLD responses for main effects are reported p<0.001 with an extent threshold of 10 contiguous voxels; BOLD= blood oxygenation level-dependent; MNI= Montreal Neurological Institute

| **Healthy controls** | | | | | | **Pathological gamblers** | | | | |
| --- | --- | --- | --- | --- | --- | --- | --- | --- | --- | --- |
|  |  | **MNI coordinates** | | |  |  | **MNI coordinates** | | |  |
|  | **L/R** | **x** | **y** | **z** | **T value** | **L/R** | **x** | **y** | **z** | **T value** |
| **Prefrontal cortex** |  |  |  |  |  |  |  |  |  |  |
| Inferior | R | 42 | 24 | 24 | 3.83 |  |  |  |  |  |
| Precentral gyrus |  |  |  |  |  | L | -36 | -15 | 66 | 3.94 |

**Postive NoGo > Neutral NoGo main effect per group.** BOLD responses for main effects are reported p<0.001 with an extent threshold of 10 contiguous voxels; BOLD= blood oxygenation level-dependent; MNI= Montreal Neurological Institute

| **Healthy controls** | | | | | | **Pathological gamblers** | | | | |
| --- | --- | --- | --- | --- | --- | --- | --- | --- | --- | --- |
|  |  | **MNI coordinates** | | |  |  | **MNI coordinates** | | |  |
|  | **L/R** | **x** | **y** | **z** | **T value** | **L/R** | **x** | **y** | **z** | **T value** |
| **Prefrontal cortex** |  |  |  |  |  |  |  |  |  |  |
| Anterior cingulate | R | 3 | 30 | 12 | 4.40 |  |  |  |  |  |
| insula | L | -39 | -12 | -3 | 3.57 | L | -45 | 9 | 6 | 4.42 |
| **Temporal lobe** |  |  |  |  |  |  |  |  |  |  |
| Hippocampus | L | -27 | -21 | -15 | 3.95 |  |  |  |  |  |

**Negative NoGo > Neutral NoGo main effect per group.** BOLD responses for main effects are reported p<0.001 with an extent threshold of 10 contiguous voxels; BOLD= blood oxygenation level-dependent; MNI= Montreal Neurological Institute
